# Case report: Significant benefits of tislelizumab combined with anlotinib in first-line treatment of metastatic renal pelvic urothelial carcinoma with sarcomatoid carcinoma differentiation

**DOI:** 10.3389/fonc.2022.969106

**Published:** 2022-10-18

**Authors:** Shibin Zhu, Chenhao Yu, Chongwei Wang, Guoqing Ding, Sheng Cheng

**Affiliations:** ^1^ Department of Urology, Sir Run Run Shaw Hospital, Zhejiang University School of Medicine, Hangzhou, China; ^2^ Department of Pathology, Sir Run Run Shaw Hospital, Zhejiang University School of Medicine, Hangzhou, China

**Keywords:** renal pelvic urothelial carcinoma, upper tract urothelial carcinoma, sarcomatoid carcinoma differentiation, tislelizumab, immune checkpoint inhibitor, tyrosine kinase inhibitor, pseudoprogression

## Abstract

**Background:**

Renal pelvic urothelial carcinoma with sarcomatoid carcinoma differentiation is a very dangerous malignant tumor and extremely rare in clinical practice. In general, these tumors with a dismal prognosis, and there is no standard treatment.

**Case presentation:**

In this case, an 81-year-old male patient was diagnosed with right renal pelvic carcinoma. After an open right radical nephroureterectomy, postoperative pathological examination showed infiltrating urothelial carcinoma with sarcomatoid differentiation. Overexpression of programmed death ligand-1 by immunohistochemistry. The carcinoma recurred 4.5 months after surgery. After informed, tislelizumab combined with anlotinib were used as first-line treatment. The patients showed a clinical partial response that lasted for 20 months.

**Conclusion:**

This case demonstrates the efficacy of tislelizumab combined with anlotinib in patients diagnosed with metastatic renal pelvic urothelial carcinoma with sarcomatoid carcinoma differentiation. Moreover, to our knowledge, this is the first application of this treatment.

## Background

Renal pelvic urothelial carcinoma with sarcomatoid carcinoma differentiation is a rare subtype of upper tract urothelial carcinoma (UTUC). As compared with pure urothelial carcinoma, UTUC with sarcomatoid differentiation are usually characterized by poor differentiation, aggressive biological behavior, and poor prognosis. Platinum-based systemic chemotherapy is recommended as first-line standard therapy for metastatic UTUC by European Association of Urology (EAU), European Society for Medical Oncology (ESMO) and National Comprehensive Cancer Network (NCCN) Guidelines (1–3). However, patients of UTUC with sarcomatoid carcinoma differentiation are not susceptible to conventional chemotherapy and have limited therapeutic options (4, 5).

Overexpression of programmed death ligand-1 (PD-L1) has been seen in patients with sarcomatoid carcinoma characteristics in recent studies (6–8), suggesting that treatment with a PD-L1/PD-1 immune checkpoint inhibitor (ICI) could be a potential option for these individuals. Two immune checkpoint inhibitors, pembrolizumab and atezolizumab, have been approved by the United States Food and Drug Administration (FDA) as a first-line treatment option for patients with locally advanced or metastatic urothelial carcinoma (mUC) who are not eligible for any platinum-containing chemotherapy (9, 10). Tislelizumab, a humanized IgG4 monoclonal antibody that targets PD-1 (11, 12), showed promising objective response rate (ORR) and overall survival (OS) in mUC. Noteworthily, around half of patients that enrolled in this study were UTUC (13). In 2020, it was approved for treatment of mUC in China. Tyrosine kinase inhibitors (TKIs) have also shown promising efficacy in multiple malignancies, and several TKI agents targeting fibroblast growth factor receptors in UC are currently being studied, with promising findings from early phase clinical trials (14). Anlotinib is a newly invented oral small molecule TKI that inhibits tumor angiogenesis and cell proliferation (15, 16). It was approved by the National Medical Products Administration (NMPA) to treat advanced non-small cell lung cancer, small cell lung cancer, soft tissue sarcoma, and thyroid cancer. However, no studies on immunotherapy or molecularly targeted pharmacological therapy for UTUC with sarcomatoid features have been published.

Here, we presented a case of recurrent renal pelvic UC with sarcomatoid carcinoma differentiation receiving tislelizumab plus anlotinib as first-line treatment. This patient achieved a clinical partial response (PR) and had a progression-free survival (PFS) up to 20 months.

## Case presentation

**Figure 1:**
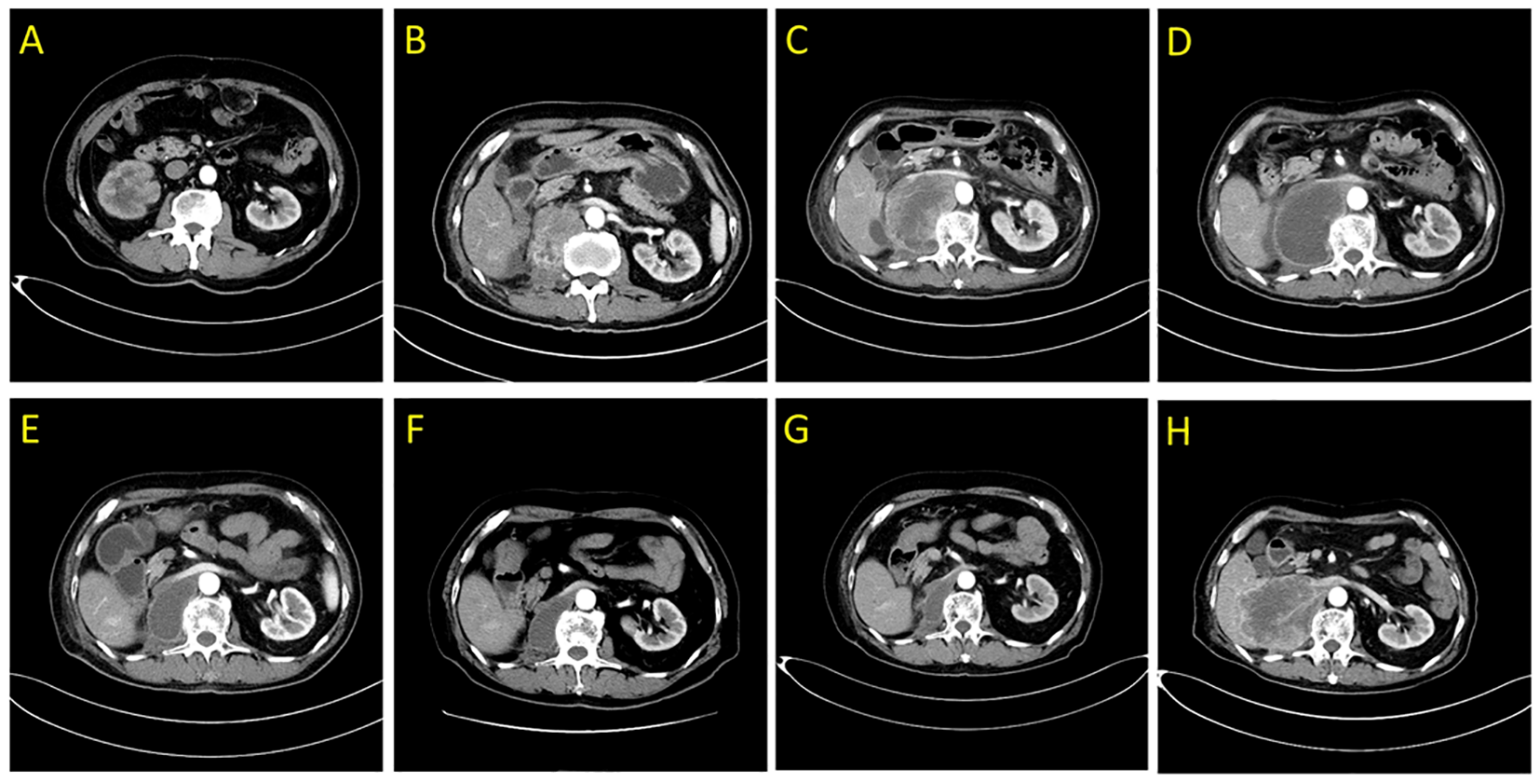
Abdominal enhanced computed tomography scans demonstrating the right posterior peritoneal tumor lesion. **(A)** at diagnosis in February 2020; **(B)** at disease relapse 4.5 months after RNU; **(C)** the tumor lesion was enlarged following 2 weeks of first dose of tislelizumab treatment in July 2020; **(D)** liquefaction and cystic changes were observed within the tumor lesion following 6 weeks of tislelizumab combined with anlotinib treatment in August 2020; **(E–G)** continuous shrinkage of the tumor lesion following tislelizumab combined with anlotinib treatment in October 2020, January 2021, and May 2021, respectively; **(H)** disease progression following 20 months of tislelizumab combined with anlotinib treatment in February 2022.

**Figure 2:**
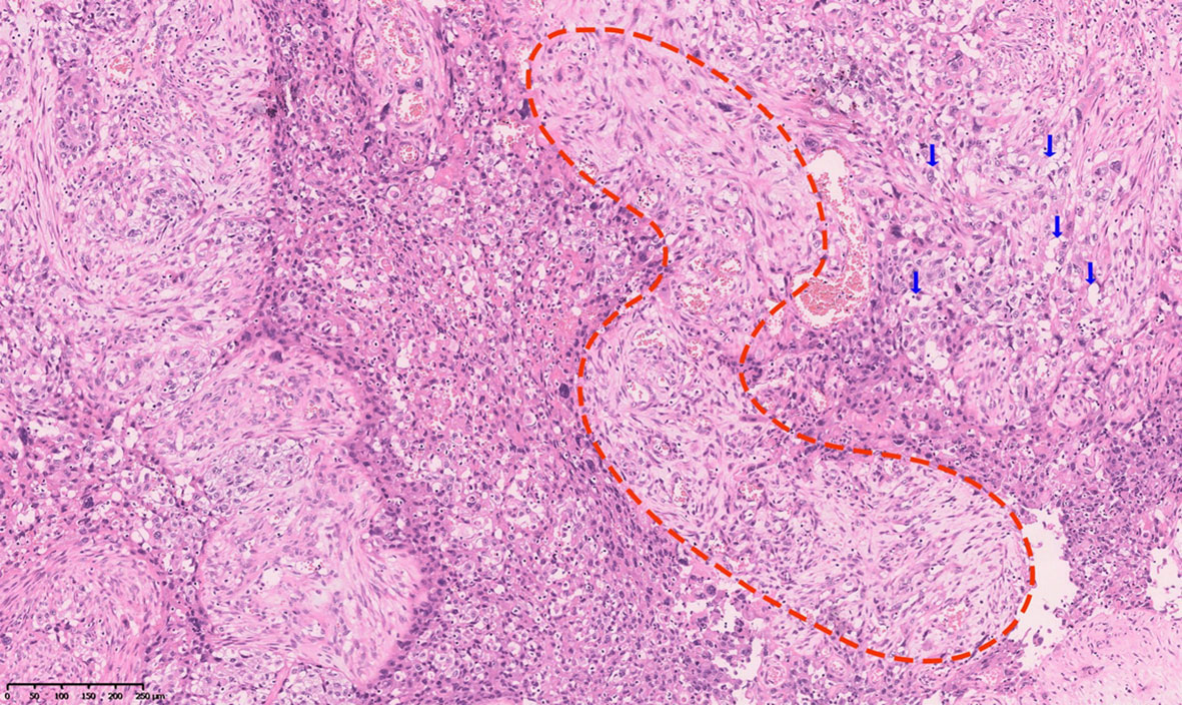
Histopathologic diagnosis: urothelial carcinoma of the right renal pelvic with sarcomatoid carcinoma differentiation. Conventional invasive high-grade urothelial carcinoma components (arrows) and sarcomatoid components (dotted circle).

An 81-year-old male patient was admitted to the hospital with right-side back pain on February 4, 2020. The abdominal enhanced computed tomography (CT) showed a 3.5 cm × 2.5 cm right renal pelvic upper mass lesion with uneven enhancement (**Figure 1A**). In urine cytology examination, nuclear atypical cells were found. The preliminary diagnosis was right renal pelvic carcinoma. The patient had undergone an open radical resection of right colon cancer nine years earlier and had a history of hypertension and taking oral calcium channel blocker (CCB) antihypertensive medication of amlodipine to control blood pressure. He had no autoimmune diseases and no long-term use of hormone drugs. One week later, an open right radical nephroureterectomy (RNU) was performed. The postoperative pathological diagnosis was infiltrating UC with sarcomatoid carcinoma differentiation (**Figure 2**). The tumor lesion size was 3.2 cm × 2.7 cm; the margin was negative; the presence of embolus in lymph vessels was detected. Immunohistochemistry (IHC) revealed positive expression of EMA, CK7, CK14, GATA-3, VM, and high PD-L1 expression (>90% of tumor cells, >1% of immune cells). Next generation sequencing (NGS) of 808-gene panel showed multiple gene mutation, including PIK3CA, KRAS, NBN, NCOR1, ARID1A, SMARCA4, PREX2, and ZFHX3. The tumor mutation burden was 12.73 mut/Mb, and the microsatellite state was microsatellite stable (MSS).

**Figure 3:**
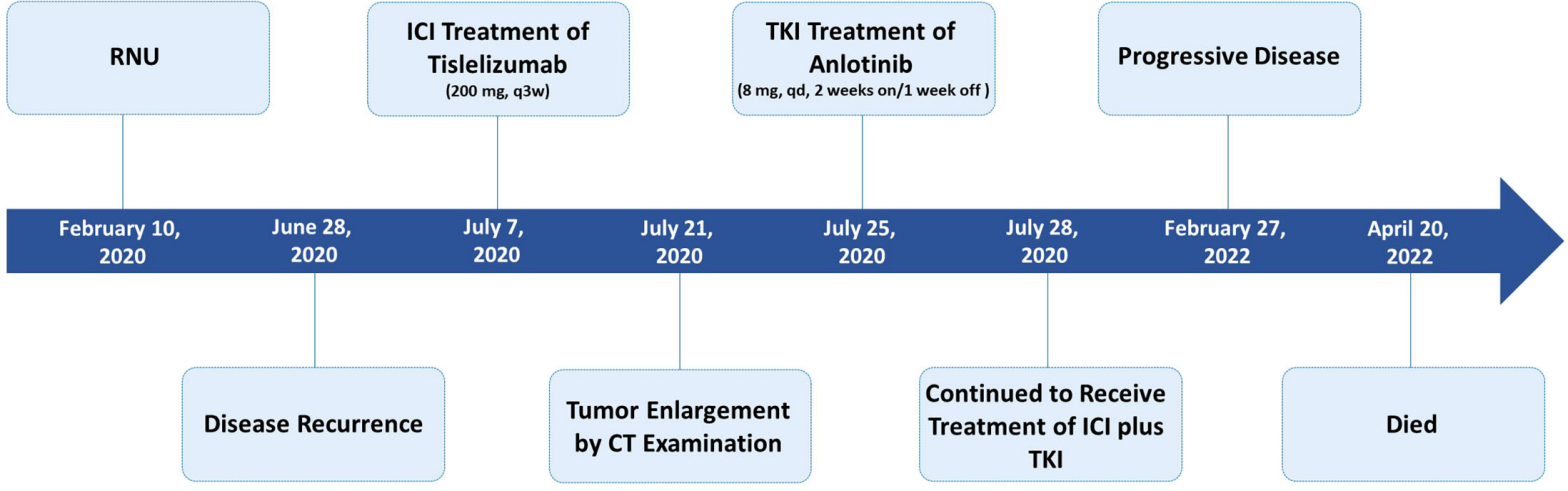
Treatment timeline.

Four and a half months after surgery, the patient felt back pain on the right side, accompanied by fatigue and poor appetite. Abdominal enhanced CT revealed renal pelvic carcinoma recurrence and invasion of the posterior peritoneum and inferior vena cava (**Figure 1B**). The ECOG score was 2 and the estimated glomerular filtration rate (eGFR) was 40.3 mL/min/1.73 m^2^. In addition, he strongly refused chemotherapy and wished to receive a therapy with favorable tolerability. Therefore, ICI (tislelizumab 200 mg, q3w) was administered after adequate informed consent on July 7, 2020. Two weeks later, the reexamination CT revealed a significant increase in the primary lesion’s tumor size (**Figure 1C**). However, the patient reported symptomatic relief from back pain and fatigue. We performed multidisciplinary-team (MDT) discussions and presumed that this patient may experience pseudoprogression but the possibility of disease progression was unneglected. Based on comprehensive consideration of the refractory sarcomatoid carcinoma differentiation and potential risk of disease progression, multi-targeting TKI (anlotinib 8 mg, qd, 2 weeks on/1 week off) treatment was added on ICI treatment after informed consent again on July 25, 2020. By the sixth week, CT scan showed significant liquefaction and cystic changes within the tumor lesion (**Figure 1D**), the patient reported continuous relief of clinical symptoms, indicating a response to ICI/TKI (tislelizumab plus anlotinib) combination therapy. Thus, he continued to receive this combined treatment. The multiple abdomen enhanced CT examinations showed a continuous shrinkage of the right posterior peritoneal tumor lesion, and the clinical response evaluation was PR (**Figures 1E–G**). The disease remained stable until February 27, 2022. In this follow-up, CT scan revealed an enlargement of the right posterior peritoneal lesion which was diagnosed as progressive disease (PD) (**Figure 1H**). No serious side effect was observed during this combined treatment and the calculated progression-free survival (PFS) was up to 20 months. Then, the patient received the best supportive care and died after developing hypercalcemia, lung infection, and respiratory failure on April 20, 2022. The treatment flowchart is shown in **Figure 3**.

## Discussion

UTUC is a rare type of tumors originated from renal calyces, renal pelvis, and ureter malignancies and accounts for 5%–7% of all kidney tumors or 5%–10% of all urothelial tumors (17). Due to the generic nature of UTUC’s clinical symptoms, missed diagnoses and misdiagnoses are prevalent, and patients are typically diagnosed at a late stage of the disease. As a result, overall prognosis of these patients is dismal. The 5-year specific survival for pT2/pT3 patients is < 50% and < 10% for pT4 patients (18). RNU is the standard of care treatment for high-risk nonmetastatic UTUC. For over three decades, platinum-based chemotherapy has been the standard first-line treatment for metastatic illness with an OS benefit of approximately 9–15 months (19). The available regimens, including MVAC (methotrexate, vinblastine, doxorubicin, and cisplatin) and GC (gemcitabine, cisplatin), show similar efficacy, although GC has a slightly better toxicity profile (20, 21).

UTUC is a disease of older patients, and many patients already have impaired renal function at the time of diagnosis (eGFR >60 in approximately 35% of patients and eGFR >45 in approximately 70% of patients), limiting their eligibility for platinum-based chemotherapy. RNU decreases the rate of eligibility for platinum-based chemotherapy to approximately 15% and 50% with a threshold of eGFR >60 and >45, respectively (22). Loss of renal function decreases eligibility for systemic chemotherapy and results in poor survival, so other effective treatment with good tolerance is essential. Immunotherapy as a new promising therapy is recommended in advanced UC by EAU/ESMO/NCCN Guidelines. Pembrolizumab and atezolizumab were approved for first-line therapy in platinum-ineligible patients by FDA based on two clinical trials. Keynote-052 is a phase 2 clinical trial that investigated the efficacy and safety of first-line pembrolizumab in cisplatin-ineligible mUC patients, including 69 (19%) UTUC patients. Approximately 24% of all patients achieved a complete response (CR) or PR, and the duration of response (DOR) was up to 33.4 months, the 4-year rate of OS was 19% (23). IMvigor210 is a multicenter, single-arm, phase 2 clinical trial that involved 119 cisplatin-ineligible patients, including 33 (28%) untreated UTUC cases. With 70.8 months of median follow-up, the observed median OS was 16.3 months and ORR was 23.5%; the higher ORR was almost 40% in patients with UTUC subgroups, and the DOR was 59.1 months in all patients (24). Therefore, first-line ICI therapy has demonstrated promising clinical efficacy in platinum-ineligible patients. In our case, the patient showed poor renal function and refused chemotherapy. After informed consent, he was administered with tislelizumab and achieved a significant and prolonged response.

UTUC with sarcomatoid carcinoma differentiation is a rare aggressive malignancy that accounts for around 7% of the histological variation of renal pelvis and ureteral malignant tumors. There are only a few examples reported in the literature (25). Sarcomatoid carcinoma has a heterogeneous, biphasic histopathologic appearance, displaying a mixture of epithelial and mesenchymal differentiation markers. It is also aggressive cancer with a terrible prognosis, as only a few individuals surviving for more than two years (26, 27). Presence of sarcomatoid histology was independently associated with shorter disease-specific survival (DSS) in comparison with conventional UC (DSS: 16 months vs. 82 months) (28). Due to the rarity of this variety, current evidence on suggested therapy and oncologic outcomes in patients with sarcomatoid features is limited, with case reports/series and retrospective cohorts providing most of the information. Intriguingly, recent research has found that PD-L1 was highly expressed in patients with sarcomatoid features, accounting for approximately 69% of pulmonary sarcomatoid carcinoma (PSC) (29) and 50% of renal sarcomatoid carcinoma (30). This observation suggested that tumor with sarcomatoid features might be sensitive to immunotherapy. In a recent case report, a PSC patient with PD-L1 overexpression achieved PR after administration of PD-1 inhibitor toripalimab, and immunotherapy combined with local radiation induced second response after progression (31). Another study showed that a PR was observed in a patient with advanced PSC and PD-L1 overexpression after the PD-1 inhibitor tislelizumab monotherapy (32).

Immunotherapy combined with TKI has also shown a promising therapeutic efficacy in tumor with sarcomatoid features. Advanced or metastasized renal cell carcinoma (mRCC) with sarcomatoid feature has a poor prognosis and is not sensitive to TKI therapy. A retrospective meta-analysis of several trials of first-line treatment with TKI in combination with ICI therapy (CheckMate 214, Keynote-426, JAVELIN Renal 101, etc.) (33) demonstrated in the sarcomatoid feature subgroup that as compared with TKI alone, superior efficacy and survival outcomes were observed in TKI/ICI combination group with ORR rates of 50–60% and a median OS of over 20 months. According to the historical data, mRCC patients with sarcomatoid feature tumors rarely survive over 12 months. The findings implied that ICI/TKI combination therapy has a clinically relevant effect on mRCC with sarcomatoid characteristics. Whether UTUC patients with sarcomatoid features can benefit from ICI monotherapy or ICI/TKI combinational therapy has not yet been reported. In our case, the patient had renal pelvic carcinoma with sarcomatoid carcinoma differentiation with high PD-L1 expression (> 90% of tumor cells, > 1% of immune cells). He achieved favorable outcome from tislelizumab plus anlotinib, and first-line PFS of 20 months was much longer than that of previous reports.

In addition to being highly effective, immunotherapy produces response patterns that differ from those seen with cytotoxic chemotherapy (34). Patients who received immunotherapy may experience a delayed response, such as temporary tumor enlargement preceded by shrinkage, stable size, or the initial emergence of tumors followed by stability or response (35). Histologically, this phenomenon can be explained as either tumor growth until a sufficient immune response develops or a transient immune cell infiltrates (36, 37). Such a response is usually asymptomatic and appropriately classified as pseudoprogression. However, the concept of pseudoprogression varies across published studies; whereas most researchers defined pseudoprogression as a PR following PD using standard RECIST criteria based on baseline tumoral burden, other authors also regarded tumoral burden stabilization after advancement (38). Interestingly, pseudoprogression may be linked to better long-term results following immunotherapy treatment, but further clinical evaluations are needed (34, 39). Pseudoprogression has become a regular occurrence in clinical practice as the grounds for immunotherapy or immune-combination therapy in cancer have expanded. However, early detection of pseudoprogression during cancer immunotherapy remains difficult, necessitating close clinical monitoring and early radiological re-evaluation. The patient in our case with renal pelvic UC showed enlargement of tumor lesions by first imaging evaluation two weeks from the first dose of tislelizumab treatment without worsening clinical symptoms. After continuing treatment of ICI-based combination therapy, imaging evaluation showed that the size of tumor lesions was significantly reduced, and the clinical response was assessed as PR, which revealed a pseudoprogression phenomenon.

## Conclusion

To the best of our knowledge, this is the first report of tislelizumab combined with anlotinib as first-line treatment in a metastatic renal pelvic UC with sarcomatoid carcinoma differentiation. The patient achieved a clinical prolonged PR for up to 20 months after a suspicious pseudoprogression phenomenon. This case indicated that tislelizumab plus anlotinib may be a promising option for these patients. However, more research is needed to understand this rare malignant tumor to optimize current treatments.

## Data availability statement

The original contributions presented in the study are included in the article/supplementary material. Further inquiries can be directed to the corresponding authors.

## Ethics statement

Written informed consent was obtained from the individual(s) for the publication of any potentially identifiable images or data included in this article.

## Author contributions

Conception/design: SC, GD. Provision of study materials or patients: SZ, SC. Collection and/or assembly of data: SZ, CW. Manuscript writing: SZ, CY. Final approval of manuscript: All authors.

## Funding

This work was supported by Medical Science and Technology Project of Zhejiang Province (2021451081); Zhejiang Medical and Health Plan Project (2020387714); Zhejiang Science and Technology Project (LGF21H160024); Wu Jieping Medical Foundation Project (320.6750.2020-14-3).

## Conflict of interest

The authors declare that the research was conducted in the absence of any commercial or financial relationships that could be construed as a potential conflict of interest.

## Publisher’s note

All claims expressed in this article are solely those of the authors and do not necessarily represent those of their affiliated organizations, or those of the publisher, the editors and the reviewers. Any product that may be evaluated in this article, or claim that may be made by its manufacturer, is not guaranteed or endorsed by the publisher.
